# Selenium Speeds Reactions

**DOI:** 10.1371/journal.pbio.0030419

**Published:** 2005-11-08

At the heart of every reaction of every cell lies an enzyme, a protein catalyst. At its active site—a special pocket on its surface—it binds reactants (substrates) and rearranges their chemical bonds, before releasing them as useful products. Rearranging some bonds may require help from certain chemical elements that are present in trace amounts. Many enzymes place these elements at the center of their active sites to do the most critical job.

Selenium is one such element. In large quantities, selenium is toxic, but, in trace amounts, it is absolutely essential for life in many organisms, including humans. Selenium is present in proteins in the form of selenocysteine, a rare amino acid that helps promote antioxidant reactions. These selenocysteine-containing proteins are called selenoproteins. One important selenoprotein is the enzyme methionine-*R*-sulfoxide reductase (MsrB) 1, whose job is to repair proteins injured by oxidative damage, caused by sunlight, toxic chemicals, or a variety of other insults.

In mammals, there are two other forms of MsrB, which also can efficiently perform this task, but use the abundant amino acid cysteine instead of selenocysteine. So why do cells go to the trouble and metabolic expense of acquiring selenium from the environment? In this issue, Hwa-Young Kim and Vadim Gladyshev explore the details of active-site chemistry of these three related enzymes, and show that the selenoprotein form employs a different catalytic mechanism.

The authors began by identifying three key amino acids in the active site of the cysteine-containing forms, which did not occur in the selenoprotein MsrB1. When any of these amino acids were mutated, the activity of the cysteine-containing enzymes was greatly diminished. This result indicates that these amino acids likely play a role at the active site, a supposition supported by previous work on related enzymes in bacteria. [Fig pbio-0030419-g001]

**Figure pbio-0030419-g001:**
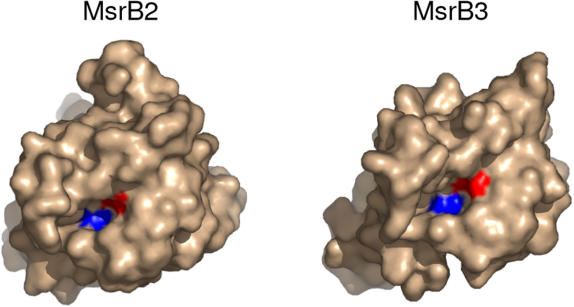
These surface models of mouse MsrB2 and human MsrB3 show the catalytic Cys95 residues in red and the Asn97 residues in blue

Kim and Gladyshev next systematically mutated MsrB1 to include one, two, or all three of these amino acids, and discovered that inclusion of one or any combination of them diminished activity of the selenocysteine-containing enzyme. This suggested that while these amino acids support the mechanism of the cysteine-containing forms, they interfere with the mechanism of the selenoprotein. Not surprisingly, when the selenium was removed from MsrB1, the enzyme was significantly impaired. But when the three amino acids were added to this crippled enzyme, they restored some of the diminished activity, probably by carrying out the same mechanism they do in the cysteine-containing enzymes.

The authors then inserted a selenium atom into each of the cysteine-containing enzymes, in the same spot in the active site where it sits in MsrB1. They found that the initial activity of each enzyme was increased over 100-fold, indicating the inherent capacity of selenium to promote catalytic activity. These souped-up enzymes were unable to complete the reaction, however, because they lacked other features of MsrB1's active site. Further scrutiny of the enzymes revealed these critical features, and inserting them allowed the artificial selenoproteins to carry out the entire reaction.

The authors suggest the explanation for these findings relates to a difference in the catalytic mechanism of selenocysteine- and cysteine-containing enzymes. The substrate for both enzyme types, methionine-*R*-sulfoxide, is found within oxidized proteins. The job of both enzymes is to reduce this compound back to the amino acid methionine. Both do so by accepting an oxygen atom from the sulfoxide.

In the presence of selenium, the oxygen temporarily binds to the selenium. The selenium's electrons then shift to bond with a sulfur on a neighboring cysteine amino acid, kicking out the oxygen as part of a water molecule. Finally, the selenium-sulfur bond is broken and the enzyme is restored to its original state by the intervention of thioredoxin, a ubiquitous cell molecule whose job is to undo just such temporary linkages in a wide variety of enzymes.

Without selenium, the oxygen binds directly to sulfur, and thioredoxin intervenes to form the water and restore the sulfur. This reaction occurs in fewer steps, but is slower. The authors propose that the evolution of selenium-containing MsrB1 from cysteine-containing forms was likely favored by the higher rate of reaction it offered, although this trend is likely limited by the requirement for changes in other portions of the enzyme to accommodate the trace element. The authors suggest that selenium provides inherent catalytic advantages to certain types of enzymatic reactions, even though utilization of these advantages is sometimes tricky. If so, manipulation of related enzymes by insertion of selenium may increase their catalytic efficiency, perhaps much above that designed by nature. This may offer advantages for some biotechnology and biomedical applications that depend on antioxidants. —*Richard Robinson*

